# Diffusion capacity of single and interconnected networks

**DOI:** 10.1038/s41467-023-37323-0

**Published:** 2023-04-18

**Authors:** Tiago A. Schieber, Laura C. Carpi, Panos M. Pardalos, Cristina Masoller, Albert Díaz-Guilera, Martín G. Ravetti

**Affiliations:** 1 Departamento de Ciências Administrativas, Universidade Federal de Minas Gerais, Belo Horizonte, MG Brazil; 2 Instituto Nacional de Ciência e Tecnologia, Sistemas Complexos, INCT-SC, CEFET-MG, Belo Horizonte, MG Brazil; 3 Machine Intelligence and Data Science Laboratory (MINDS), Universidade Federal de Minas Gerais, Belo Horizonte, MG Brazil; 4 Industrial and Systems Engineering, University of Florida, Gainesville, FL USA; 5 Lab LATNA, National Research University, Higher School of Economics, Nizhny Novgorod, Russia; 6 Departament de Física, Universitat Politècnica de Catalunya, Terrassa, BCN Spain; 7 Departament de Física de la Matèria Condensada, Universitat de Barcelona, Barcelona, BCN Spain; 8 Universitat de Barcelona Institute of Complex Systems (UBICS), Universitat de Barcelona, Barcelona, BCN Spain; 9 Departamento de Ciência da Computação, Universidade Federal de Minas Gerais, Belo Horizonte, MG Brazil

**Keywords:** Complex networks, Nonlinear phenomena, Information theory and computation

## Abstract

Understanding diffusive processes in networks is a significant challenge in complexity science. Networks possess a diffusive potential that depends on their topological configuration, but diffusion also relies on the process and initial conditions. This article presents Diffusion Capacity, a concept that measures a node’s potential to diffuse information based on a distance distribution that considers both geodesic and weighted shortest paths and dynamical features of the diffusion process. Diffusion Capacity thoroughly describes the role of individual nodes during a diffusion process and can identify structural modifications that may improve diffusion mechanisms. The article defines Diffusion Capacity for interconnected networks and introduces Relative Gain, which compares the performance of a node in a single structure versus an interconnected one. The method applies to a global climate network constructed from surface air temperature data, revealing a significant change in diffusion capacity around the year 2000, suggesting a loss of the planet’s diffusion capacity that could contribute to the emergence of more frequent climatic events.

## Abstract (web summary)

Understanding of diffusive and spreading processes in networks remains challenging when dynamics of the network is complex. The authors propose a quantity to reflect the potential of a network node to diffuse information, that may serve to develop interventions for improved network efficiency.

## Introduction

Natural and artificial diffusive processes from the most varied contexts are omnipresent in our everyday lives^1–6^. Advancing our understanding of diffusive processes is a fundamental challenge with critical practical applications across a wide range of spatial scales. For instance, diffusion magnetic resonance is an imaging technique that allows studying the brain’s structural and functional connectivity^7,8^. A diffusion-like process describes the action of infectious agents that attack our immune system spreading as fast as they can^9^. On a large scale, the billions of individuals commuting daily between different geographical regions constitute the highly complex global human mobility system^10–12^. Similarly, gossip spreads through vast complex social networks^3,4,13–17^. All these phenomena have motivated researchers to understand the mechanisms that enhance or suppress diffusion, and to quantify their impacts^2,18–21^.

Structural and dynamical properties of diffusion processes have been successfully modeled by networks, structures that are able to encompass this complex combination^2,18–28^. The analysis of diffusive processes usually assumes that interaction networks represent their average behavior^18^; some models consider random navigation^29–34^, and others consider the topological shortest paths^35^. Shortest paths are essential for diffusion processes since they are the ones through which nodes are firstly reached when a process starts diffusing in a structure in which connections are of the same nature. However, geodesical paths are not the only ones that need to be considered, as the characteristics of the connections interfere in a way that could either improve or worsen the transmission of information. Topological features can be considered as connection weights to define the weighted shortest paths. Then, the inclusion of both, geodesical and weighted shortest paths could lead a more precise evaluation of the outcome of the diffusion process^34,36–38^.

The influence of specific topological features, such as community structures or degree heterogeneity of nodes on diffusion processes, has been described in several works^39–41^. However, one interesting point that still needs to be addressed in the literature is the study of the way individual nodes change their performance as diffusing agents during the evolution of the diffusive process. This performance depends not only on the node’s structural connectivity but also on the dynamic process on top of the structure and initial conditions. Considering that nodes change the way they diffuse as the process evolves, we propose a new measure called Diffusion Capacity that is able to track the evolution of the node’s performance. Diffusion-Capacity quantifies the evolving diffusive ability of nodes through the use of a weighted distance distribution that allows the inclusion of dynamical features of the process. We introduce in this work a method that brings many possibilities for strategic interventions to design more efficient diffusive structures.

The concept of Diffusion Capacity is also extended to interconnected networks, structures that represent a system composed by several networks interacting simultaneously. These systems are a good representation of natural systems since they are the result of the complex interaction of many subsystems. Considering this we propose a measure called Relative Gain (${{{{{{{\mathcal{G}}}}}}}}$) that compares the diffusive performance of an element acting in an isolated network and its performance when it is part of an interconnected structure. Among the examples presented in this work we consider heat diffusion, Kuramoto oscillators, disease spreading and a climate network. This variety of applications reflects the versatility of the measures here proposed and their efficacy in revealing useful diffusive features.

## Results

### Single networks

#### Climate networks

**Fig. 1:**
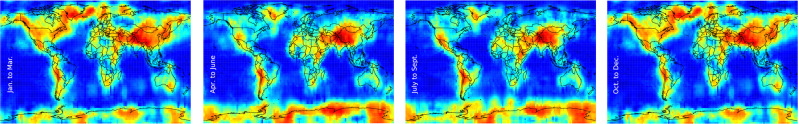
Spatial patterns of diffusion-capacity values that correspond to average values of trimestral networks. Colors of geographical points correspond to the diffusion-capacity mean value of each season computed through daily surface air temperature data.

As a real world example of heat diffusion we use the gridded reanalysis dataset of Surface Air Temperature (SAT)^42^ to analyze the way the diffusion-capacity measure captures the heat flow through the Earth surface. For this example diffusion capacity is computed for each geographical point (2.5 latitude × 2.5 longitude) of a gridded Earth network in which the weights of the connections are the temperature differences between points. Trimestral means of node diffusion-capacity values are depicted in Fig. 1. Some interesting global features can be observed from the diffusion-capacity spatial patterns. In general, superficial air over oceans show lower diffusion-capacity values than superficial air over land, increasing gradually in coastal waters. Considering only land areas, higher diffusion-capacity values are observed in places of higher altitudes as well as in winters of colder places (see extratropical places in South Hemisphere in Fig. 1B, C, and extratropical places in North Hemisphere in Fig. 1A, D). Another interesting fact is that diffusion-capacity variation among seasons is much lower between tropics than in extratropical regions.

**Fig. 2:**
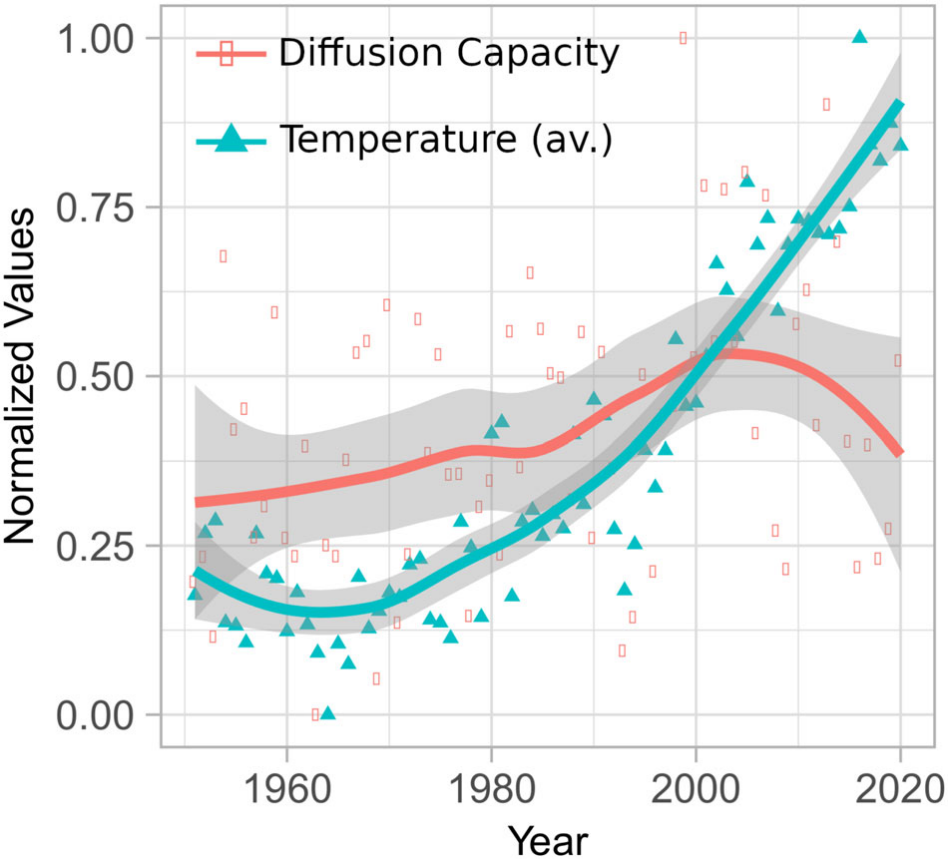
Comparison of Annual Diffusion Capacity and Temperature Values in Global Surface Air Temperature Climate Network. Annual values of diffusion Capacity (orange) and annual temperature values (green) for the global surface air temperature (SAT) climate network.

Figure 2 depicts the average global temperature and global diffusion-capacity values from 1951 to 2020. It is possible to see that while temperatures rise constantly, diffusion-capacity drastically changes its tendency around 2000. It is worth mentioning, that extreme weather events has increased significantly in the last 20 years almost duplicating the number of natural disasters^43–46^.

#### Disease spreading

**Fig. 3:**
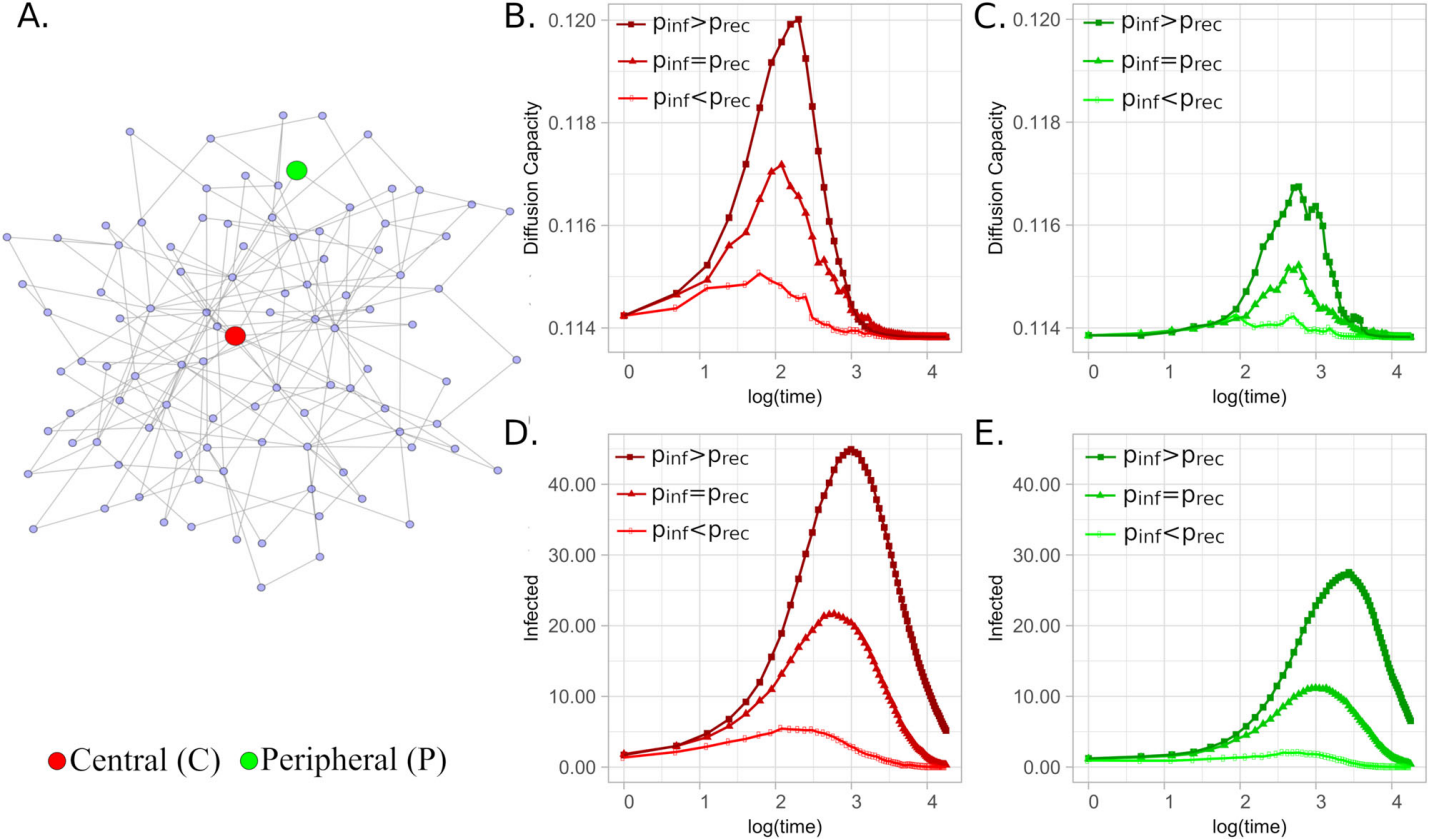
Diffusion Capacity Analysis of SIR Models with different initial conditions. Small network highlighting a central (red) and a peripheral (green) node (**A**). Network diffusion-capacity evolution of a SIR model when the central node (**B**) and a peripheral node (**C**) is initially infected, for three different infection probabilities. **D**, **E** depict the evolution of the number of infections respectively. All figures show mean values of 100 realization considering a constant *p*_*inf*_ = 0.1 and *p*_*rec*_ = 0.05, 0.10 and 0.20.

The SIR model^47–50^ is a simple mathematical model for the spread of epidemic diseases, in which, nodes can be susceptible, infected, or recovered from an infectious agent. Recovered nodes are immune to the disease, while a susceptible node can become infected if it is in contact with an infected node. We study here, the evolution of the SIR model in a small network (Fig. 3A) when one central node is initially infected, and also when a peripheral node initiates the epidemic process. In this experiment, we consider the probability of a susceptible node to become infected is *p*_*inf*_ = 0.1 and different recovery rates *p*_*rec*_ values.

In general, when the process begins with the infection of a central node, the number of new infections grows more and faster, reaching earlier immunity, than when the process is initiated in a peripheral node. In this case, the number of new infected nodes grows less and slower, having its maximum later. The evolution of the dynamic also depends on the probability of recovery (*p*_*rec*_) of infected nodes, that induces different outcomes.

In Fig. 3 it is depicted a SIR model when the probability of infection is greater than the probability of recovery (*p*_*inf*_ > *p*_*rec*_), when the probabilities are equal (*p*_*inf*_ = *p*_*rec*_), and finally when the probability of infection is lower than the probability of recovery (*p*_*inf*_ < *p*_*rec*_). The lower the probability of recovery, the faster the disease spreads. Figure 3B, C show the evolution of the diffusion capacity when the central, and peripheral nodes are initially infected for different recovery probability values. For *p*_*inf*_ > *p*_*rec*_ values of diffusion capacity present the highest peak, lower for the process initiated in the peripheral node. The lowest values correspond to the case of *p*_*rec*_ > *p*_*inf*_.

Figure 3D, E compare the different infection processes explained above. These figures show that the initial diffusion capacity is higher when the process is initiated in the central node. As the process evolves, the diffusion capacity increases until a maximum that appears earlier than the maximum number of cases, showing itself as an early indicator of the peak of the epidemic process. As in this case, weights are the same in all links, peripheral nodes posses a lower diffusion capacity than central nodes, then they are more affected by changes in the dynamical process. As central nodes already are strong diffusive agents, the impact of changes is less significant. This information can be useful to plan strategies to reduce the impact of these kind of spreading diseases.

### Interconnected networks

#### Kuramoto oscillators

**Fig. 4:**
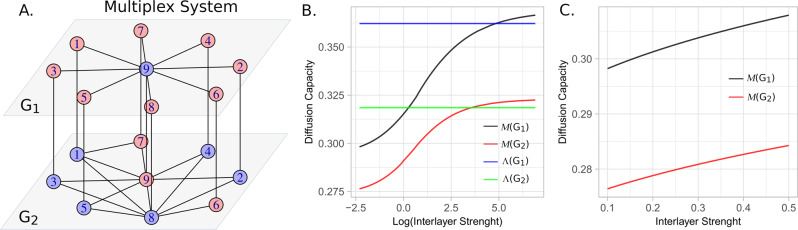
Multiplex network formed by layers *G*_1_ and *G*_2_ connected by weighted links with equal interlayer strength. Nodes in blue/red represent those that decrease/increase their diffusion capacity in the fully coupled multilayer structure, compared to their diffusion capacity in isolation (**A**). Diffusion Capacity Λ of *G*_1_, *G*_2_ and, for different interlayer strengths (logarithmic scale) the multilayer diffusion capacity ${{{{{{{\mathcal{M}}}}}}}}({G}_{1})$ and ${{{{{{{\mathcal{M}}}}}}}}({G}_{2})$ (**B**). Evolution of the Multilayer diffusion Capacity for small interlayer strength values (**C**).

**Fig. 5:**
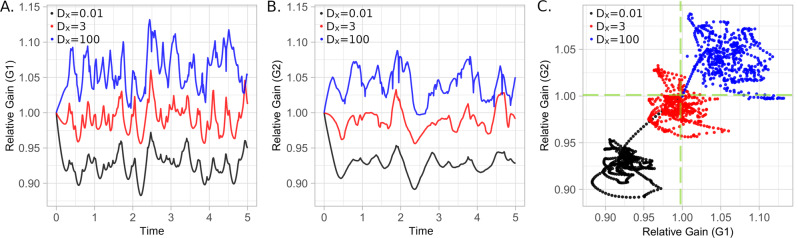
Time evolution of a random experiment for Kuramoto’s model (Eq. 1) on the multiplex system of Fig. 4A in which, initially, all oscillators are in phase (*θ*_*i*_ = 0) having their fundamental frequencies chosen from a uniform distribution [0, 1] for different coupling constants *D*_*x*_. **A**, **B** Shows the evolution of the relative gains of layers *G*_1_ and *G*_2_, respectively, and (**C**) the plane ${{{{{{{{\mathcal{G}}}}}}}}}_{1}\,\times\, {{{{{{{{\mathcal{G}}}}}}}}}_{2}$ for the three different coupling constants considered. In all experiments the value of *c* is 0.01/18.

To understand the meaning of the relative gain in a different dynamic, we consider the well-known Kuramoto’s model^51–54^ in the multiplex system of Fig. 4A. Coupled by a constant *D*_*x*_, for each vertex $i\in \overrightarrow{G}$ there is an oscillator with phase *θ*_*i*_ that varies following the differential equation for any node *i* belonging to a generic layer *L*:

1 $${\dot{\theta }}_{i}\,=\,{\omega }_{i}\,+\,c\left[\mathop{\sum}\limits_{j\in {V}_{L}}{A}_{i,j}sin({\theta }_{j}\,-\,{\theta }_{i})\,+\,\mathop{\sum}\limits_{k\notin {V}_{L}}{A}_{i,k}{D}_{x}sin({\theta }_{k}\,-\,{\theta }_{i})\right],$$

where, *ω*_*i*_ is the fundamental frequency of the oscillator *i*, *c* a constant, and *A* the adjacency matrix of the multilayered system. Figure 5A, B shows the corresponding relative gains ${{{{{{{{\mathcal{G}}}}}}}}}_{L}$ of each isolated layer for three different coupling constants.

It is possible to see that, for *D*_*x*_ = 0.01 the relative gains of both layers are less than one, and this means that the shortest path between two nodes that belong to the same layer, is in the layer. When *D*_*x*_ = 100 the relative gains of both layers are higher than one showing that shortest paths between pairs of nodes in one layer use connections in the other. For *D*_*x*_ = 3, the relative gain presents an interesting behavior being less than 1 in some periods as for *D*_*x*_ = 0.01 regime, and greater than 1 in other periods as in *D*_*x*_ = 100. Additional information regarding DC is presented in the last three sections of the Supplementary Information. Degree correlations between layers are analyzed in Section G, and a multilayer brain network to study different hearing pathologies is presented in Section H.

## Discussion

In this study, we present the concept of Diffusion Capacity, a quantity that offers insights into a node’s ability to diffuse information. By leveraging a probability distribution that encodes information about geodesic and weighted paths, we define Diffusion Capacity and propose a method for computing it. Our approach allows for the inclusion of dynamical characteristics of the diffusion process, providing local and global temporal information about the system’s performance.

We extend the concept of Diffusion Capacity to interconnected networks, enabling us to quantify changes in the node’s diffusive performance when it is connected to other structures. We introduce a new quantity, called Relative Gain, which measures the change in Diffusion Capacity of a node due to the inclusion of new networks into the system. This approach allows to project superdiffusive structures, as well as enhance or suppress diffusion by modifying the network’s topology.

As a real data application, we study the temporal evolution of Diffusion Capacity in a global climate network constructed using SAT data. Our findings reveal a significant change in tendency around the year 2000, despite the continuous rising of global temperatures. This result suggests a loss of the planet’s Diffusion Capacity, which may be a contributing factor to the emergence of more frequent and extreme climatic events. Further research is necessary to investigate this finding and its implications for the planet’s climate.

In conclusion, our work introduces an approach to quantify the Diffusion Capacity of nodes in a network and identify potential interventions for improving the system’s efficiency. We believe that our approach has broad applications in various fields, including transportation, social networks, and epidemiology, among others.

## Methods

### Paths in single networks

**Fig. 6:**
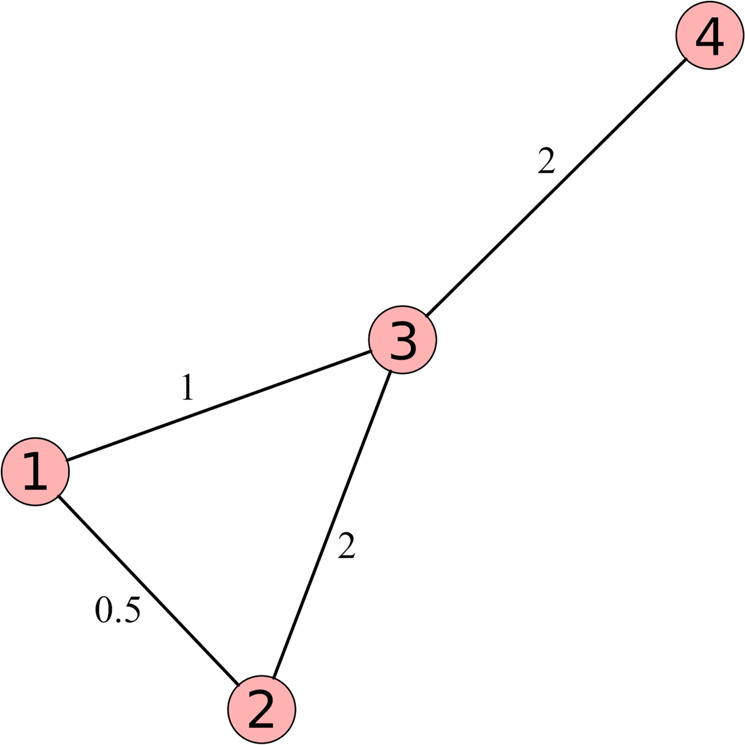
Example of a network composed of 4 nodes and 4 links, with different values of topological weights. The weights reflect the strength of the connections between nodes.

By definition, the geodesic distance between nodes *i* and *j*, *D*_*g*_(*i*, *j*) is the minimum number of links separating them. The weighted distance between nodes *i* and *j*, *D*_*w*_(*i*, *j*), is the minimum sum of the inverse of the weights connecting both nodes. Then, considering Fig. 6, the geodesical path between nodes 1 and 2 is 1 → 2, then *D*_*g*_ = 1. The weighted path between nodes 1 and 2 is 1 → 3 → 2, as the corresponding weights are 1 (*D*_*w*_ = 1) for link 1 → 3 and 2 (*D*_*w*_ = 1/2) for link 3 → 2, then *D*_*w*_ = 1 + 1/2 = 3/2.

*D*_*g*_(*i*, *j*) and *D*_*w*_(*i*, *j*) allow the proposition of a weighted node distance distribution (wNDD) based on the quantity Δ_*i*,*j*_ = *D*_*w*_(*i*, *j*)/*D*_*g*_(*i*, *j*), the ratio of the weighted and geodesical distances between nodes *i* and *j*^55–57^.

If Δ_*i*,*j*_ < 1 the weighted distance connecting *i* to *j* is smaller than the geodesical distance (*D*_*g*_ > *D*_*w*_), and 1 − Δ_*i*,*j*_ quantifies this difference. If Δ_*i*,*j*_ > 1, the weighted distance connecting *i* to *j* is larger than the geodesical distance *D*_*g*_(*i*, *j*) < *D*_*w*_(*i*, *j*), and this difference can be quantified by 1 − 1/Δ_*i*,*j*_.

Considering a network G=(V,E), where V is the set of nodes and E is the set of edges, for each *i* ∈ *V* and *d* = 1, 2…∣*V*∣ − 1, *∞*, let Γ_*i*_(*d*) = {*y* ∈ *V*∣*D*_*g*_(*i*, *j*) = *d*}. The fraction of disconnected nodes from *i* is defined as **p**_*i*_(*∞*) = ∣Γ_*i*_(*∞*)∣/(∣**V**∣ − 1), and the fraction of nodes at geodesic distance *d* from *i* for *d* ≠ *∞*, **p**_*i*_(*d*) = ∣Γ_*i*_(*d*)∣/(∣**V**∣ − 1) as the vector:

$$\left[{{{{{{{{\bf{p}}}}}}}}}^{+}i(d),\,{{{{{{{{\bf{p}}}}}}}}}_{i}^{0}(d),\,{{{{{{{{\bf{p}}}}}}}}}^{-}i(d)\right]\,=\,\frac{1}{|V|-1}\mathop{\sum}\limits_{y\in {\Gamma }_{i}(d)}\left[\max (1\,-\,{\Delta }_{i,j},\,0),\min \left({\Delta }_{i,j},\frac{1}{{\Delta }_{i,j}}\right),\max \left(1\,-\,\frac{1}{{\Delta }_{i,j}},0\right)\right]$$

in which, ${{{{{{{{\bf{p}}}}}}}}}_{i}^{+}(d)$ is the fraction of nodes at geodesic distance *d* from *i*, for which their weighted shortest path is smaller than $d,\,{{{{{{{{\bf{p}}}}}}}}}_{i}^{-}(d)$ indicates the fraction of nodes at geodesic distance *d* from *i*, for which their weighted shortest path is larger than *d*, and ${{{{{{{{\bf{p}}}}}}}}}_{i}^{0}(d)$ is the fraction of nodes for which there is no difference between the paths. Considering this, if all weights are one, ${{{{{{{{\bf{p}}}}}}}}}_{i}^{+}(d)\,=\,0$ and ${{{{{{{{\bf{p}}}}}}}}}_{i}^{-}(d)\,=\,0$. In the extreme case that weights tend to infinity \, and ${{{{{{{{\bf{p}}}}}}}}}_{i}^{0}(d)\to 0$, being the topological configuration, irrelevant. On the other hand, if weights tend to zero, ${{{{{{{{\bf{p}}}}}}}}}_{i}^{-}(d)\to|{\Gamma }_{i}(d)|/(|V|-1),{{{{{{{{\bf{p}}}}}}}}}_{i}^{+}(d)=0$ and ${{{{{{{{\bf{p}}}}}}}}}_{i}^{0}(d)\,\to\, 0$, approximating to the situation of considering only the geodesic distances. The weighted node distance distribution wNDD is then defined for each node, as:

2 $${{\mathbb{P}}}_{i}\,=\,\left[{{{{{{{{\bf{p}}}}}}}}}_{i}^{+}(1),\,{{{{{{{{\bf{p}}}}}}}}}_{i}^{0}(1),\,{{{{{{{{\bf{p}}}}}}}}}_{i}^{-}(1),\ldots,\,{{{{{{{{\bf{p}}}}}}}}}_{i}^{+}(|V|-1),\,{{{{{{{{\bf{p}}}}}}}}}_{i}^{0}(|V|-1),\,{{{{{{{{\bf{p}}}}}}}}}_{i}^{-}(|V|-1),\,{{{{{{{{\bf{p}}}}}}}}}_{i}(\infty )\right],$$

${{\mathbb{P}}}_{i}\,=\,(1,\,0,\,0,\ldots,\,0)$ is the form of the distance distribution (wNDD) of a node in a fully connected network with weights tending to infinite. When larger weights accelerate the diffusion process, ${{\mathbb{P}}}_{i}$ corresponds to the distance distribution of a node in the most diffusive structure, and for that, we consider them as our reference structure *G*_*ref*_ and distribution ${{\mathbb{P}}}_{ref}$.

### Diffusion Capacity in single networks

Then, let *G* = (*V*, *E*, *W*) be a weighted network composed by a set of vertices V, edges E and weights W. We define the diffusion capacity of node *i*, Λ_*i*_(*G*), as the inverse of the distance between ${{\mathbb{P}}}_{i}(G)$ and ${{\mathbb{P}}}_{i}({G}_{ref})$, where *G*_*ref*_ is the graph reference of the same size than *G*. We measure the distance between these distributions using the cumulative Jensen-Shannon divergence (CDD), described in Section A of the Supplementary Information (SI). Therefore, the Diffusion Capacity (DC) of node *i* ∈ *G*, Λ_*i*_(*G*), is

3 $${\Lambda }_{i}(G)\,=\,{\left[CDD({{\mathbb{P}}}_{i},\,{{\mathbb{P}}}_{ref})\right]}^{-1}$$

and the diffusion capacity of the network *G*, Λ_*D*_(*G*), is the average over all nodes

4 $$\Lambda (G)\,=\,\frac{1}{|V|}\mathop{\sum}\limits_{i\in V}{\Lambda }_{i}(G).$$

**Fig. 7:**
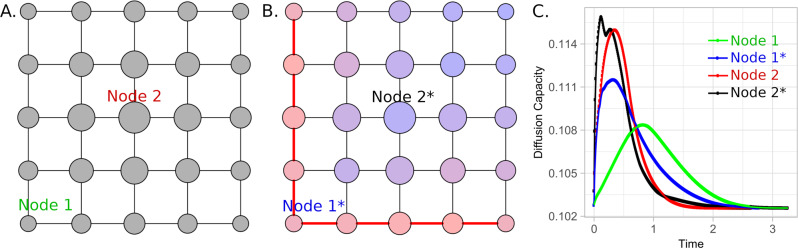
Regular grid in which the size of the nodes correspond to their topological diffusion capacity. **A** and the topological diffusion capacity when some peripheral links possess a higher weight (**B**). Two different heat diffusion processes are initiated in nodes 1 or 2 in both structures by assigning a temperature 25 degrees higher than the temperature of the other nodes. The time evolution of the diffusion capacity values of the system is shown in (**C**). The minimum value (DC_*min*_) corresponds to the DC value when (*x*_*j*_ − *x*_*i*_) → 0.

To study diffusion processes in networks we propose the embedding of dynamical features in the topological weights. Then, weights considered in this analysis are the result of the combination of topological and dynamical characteristics of the structure and process, respectively. Figure 7 depicts a simple example of a heat diffusion process on a regular network^58^. In this case, the weights considered in the analysis are the combination of the thermal conductivity of the links *w*_*i*,*j*_ (topological weights), and the temperature difference between the nodes involved (*x*_*j*_ − *x*_*i*_) (dynamical feature), *W*_*i*,*j*_ = *w*_*i*,*j*_(*x*_*j*_ − *x*_*i*_) as explained in Section B of the SI. Nodes interact by exchanging heat with a rate proportional to their temperature difference and to the thermal conductivity. In thermal equilibrium, as there is no energy flow, the diffusion capacity (DC) of nodes, represented by the node’s sizes, depends exclusively on the topological structure. However, when energy flows, the DC of the nodes varies until reaching equilibrium. The system’s DC, defined as the average of the node’s DC, increases to a maximum value and returns to the state in which no flow is present. In Fig. 7A the thermal conductivity is the same in all connections, then, because of their global topological configuration, central nodes have higher DC values. In Fig. 7B the thermal conductivity of some connections is increased, then, the size of the nodes also increase in different proportions depending of their topological location. To better analyze this process, we simulate the evolution of the heat exchange and compute, at each time step, the DC value of the whole structure as depicted in Fig. 7C. First, we increase the temperature of node 1 and compute DC in structure A, as shown by the green and blue lines. By comparing these processes, it is possible to see that, under the same initial conditions, the system in B reaches higher DC values and reaches thermal equilibrium in a shorter time than the system in A. However, after increasing the temperature of node 2 and computing DC in structures A and B, as shown by the red and black lines, respectively, contrary to what it is expected because of the presence of some connections with higher thermal conductivity in structure B, the process in structure A reaches thermal equilibrium earlier.

Two important DC values exist for every system. By assuming (*x*_*j*_ − *x*_*i*_) = 1 as an arbitrary value, we have information about the relative DC values between nodes to represent their structural latent diffusive potential. This value corresponds to the topological diffusion capacity (DC_*t*_). Figure 7 shows two regular grids, in which DC_*t*_ values are represented by the size of the nodes when all connections have the same weight (A) and when some links possess a higher thermal conductivity (thicker lines) in (B). The color scale in Fig. 7B represents the percentage increase in DC_*t*_ considering only the topological change.

The second important network DC value is the asymptotic minimum depicted in Fig. 7C, where all DC curves converge in different moments, independently of the weights of the connections, the dynamical process involved and the initial conditions. This minimum DC value (DC_*min*_) is unique for each specific adjacency matrix, and corresponds to the case in which (*x*_*j*_ − *x*_*i*_) → 0, and only geodesic distances count.

Section C of the SI presents an experiment of DC applied to Kuramoto oscillators, and the computational complexity of DC is discussed in Section D.

### Paths in interconnected networks

**Fig. 8:**
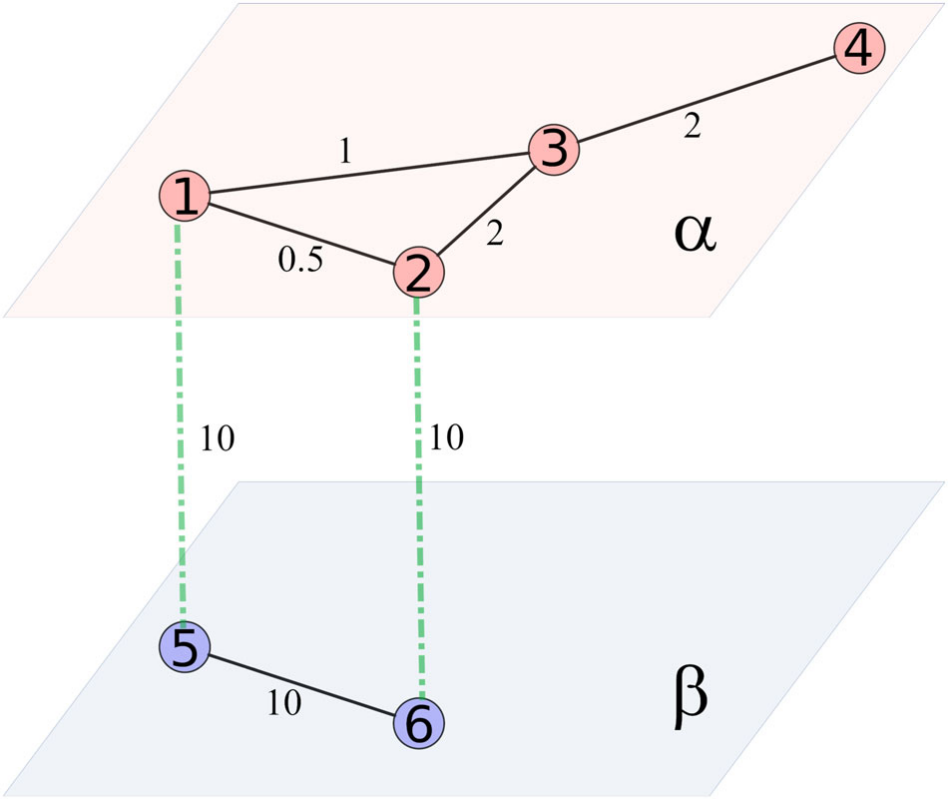
Bilayer network composed by layer *α* possessing 4 nodes and layer *β* possessing 2 nodes. Layers *α* and *β* are interconnected through nodes by a diffusion coefficient *D*_*x*_ = 10.

When considering interconnected networks, represented as multilayer structures, we have to consider paths using more than one layer. Using the concepts of single and doubly connected nodes proposed in the Lace Expansion method^59^, we define that two nodes *i* and *j*, of the same layer, are doubly connected (*i* ⇔ *j*) if it exists, between them, at least a shortest path going through two different layers. Then, two nodes are single connected (*i* ↔ *j*) if they are not doubly connected. For example, vertices 1 and 3 of Fig. 8 are doubly connected because the shortest path connecting them contains links in more than one layer (1–5–6–3). Vertices 5 and 6, on the other hand, are not doubly connected because the shortest path connecting them corresponds to the same layer. Then, the wNDD of a multilayer system have to consider the intralayer links.

For a weighted multilayer network composed by a set of *M* weighted networks, $\overrightarrow{G}\,=\,({G}_{1},\,{G}_{2},\ldots,{G}_{M},\,{\mathbb{E}})$ with *G*_*L*_ = (*V*_*L*_, *E*_*L*_, *W*_*L*_), *L* = 1, 2, …, *M*, and layers connected by weighted interlayer links ${\mathbb{E}}=\,\{{w}_{x,y}|x\,\in\, {V}_{\alpha },y\,\in\, {V}_{\beta },\alpha \,\ne\, \beta \}$, we define for nodes *i*, *j* ∈ *V*_*L*_:

1. *D*_*w*_(*i*, *j*), as the shortest distance over all paths connecting *i* and *j* in the multilayered system, being ${{\mathbb{P}}}_{i}$ its corresponding probability distribution;
2. ${D}_{w}^{\beta }(i,j)$, the shortest distance over all paths connecting *i* and *j* such that at least one node in the path belongs to a different layer, *G*_*β*_ with *β* ≠ *L*, being ${{\mathbb{P}}}_{i}^{\beta }$ its corresponding probability distribution.

For every $i\in {V}_{L},{{\mathbb{P}}}_{i}$ captures the impact of the shortest paths reduction due to the multilayer structure, and ${{\mathbb{P}}}_{i}^{\beta }$ captures the impact of the paths that are forced to have, at least, one link connected to layer *G*_*β*_. In the SI section E we present, in detail, the construction of the probability distributions for the network depicted in Fig. 8.

### Diffusion capacity in interconnected networks

To define DC we use ${{\mathbb{P}}}_{ref}$ that, in this case, corresponds to the most diffusive multilayer structure. In these structures, if there are no interlayer connections, diffusion occurs independently in each layer. However, when the interlayer strength is low, the diffusion time may become excessively long, as these weak interlayer connections slow down the dynamics of both layers. On the other hand, a strong interaction between layers enhances diffusion.

Let $\overrightarrow{G}\,=\,({G}_{1},\,{G}_{2},\ldots,{G}_{M},\,{\mathbb{E}})$ a multilayer weighted network, we define, for each node *i* ∈ *V*_*L*_ and for all *L* = 1, 2, …, *M*:

1. The Node Diffusion Capacity (${{{{{{{{\mathcal{M}}}}}}}}}_{i}$):
  5 $${{{{{{{{\mathcal{M}}}}}}}}}_{i}(\overrightarrow{G})\,=\,{\left[\frac{1}{2}CDD({{\mathbb{P}}}_{i},\,{{\mathbb{P}}}_{ref})\,+\,\frac{1}{2M-2}\mathop{\sum }\limits_{\beta=1,\,\beta \,\ne\, L}^{M}CDD({{\mathbb{P}}}_{i}^{\beta },\,{{\mathbb{P}}}_{ref})\right]}^{-1}$$
2. The Layer Diffusion Capacity is defined as the average of ${{{{{{{{\mathcal{M}}}}}}}}}_{i}$ over all the nodes in a layer,
  6 $${{{{{{{\mathcal{M}}}}}}}}({G}_{L})\,=\,\frac{1}{|{V}_{L}|}\mathop{\sum}\limits_{i\,\in\, {V}_{L}}{{{{{{{{\mathcal{M}}}}}}}}}_{i}$$

In the first term of Eq. 5, ${{\mathbb{P}}}_{i}$ is the distribution of the multilayer dynamical paths between node *i* and the other nodes of layer *G*_*L*_ (see methods). $CDD({{\mathbb{P}}}_{i},{{\mathbb{P}}}_{ref})$ compares the diffusive potential of a node through a distance to a reference distribution. In the second term, ${{\mathbb{P}}}_{i}^{\beta }$ represents the distribution of the multilayer paths between node *i* and the other nodes of layer *G*_*L*_, for which paths are imposed to go through layer *β* (see methods). The average of $CDD({{\mathbb{P}}}_{i}^{\beta },\,{{\mathbb{P}}}_{ref})$ captures the effect caused by the presence of all *β* ≠ *L*, on node *i*. In this way, the multilayer DC of node $i,\,{{{{{{{{\mathcal{M}}}}}}}}}_{i}(\overrightarrow{G})$ is represented by the structural and dynamical dissimilarity between the multilayer connectivity of node *i* in *G*, and the multilayer connectivity of node *i* in *G*_*ref*_.

#### Relative gain

Figure 4 shows a multiplex system containing nine nodes in each layer, coupled through a constant interlayer weight between layers *G*_1_ and *G*_2_ (Fig. 4A). Multiplex networks are multilayer structures with the restriction of having the same set of nodes in all layers, and intralayer connections exclusively through the same nodes. Figure 4B depicts the DC values of isolated layers (Λ(*G*_1_) and Λ(*G*_2_), and for the multiplex system, (${{{{{{{\mathcal{M}}}}}}}}(G1)$ and ${{{{{{{\mathcal{M}}}}}}}}(G2)$), for different interlayer weights. In this system, as the coupling increases, the diffusion capacities of the multilayer system also increase up to a point where ${{{{{{{\mathcal{M}}}}}}}}(G1) \, > \,\Lambda (G1)$ and ${{{{{{{\mathcal{M}}}}}}}}(G2) \, > \,\Lambda (G2)$. Figure 4C shows that for small values of the coupling constant, the increase in diffusion capacity is nearly linear. DC values can be found in Table 1. This result is consistent with previous findings in^60^ in which it is shown that the time for a multiplex network to reach equilibrium, scales with the inverse of the smallest positive eigenvalue of the Laplacian matrix (*λ*_2_). In the Section F of the SI we show the relationship between DC and *λ*_2_ values.

**Table 1:** Mono and multilayer diffusion-capacity values for the system depicted in Fig. 4

	Nodes									
DC	1	2	3	4	5	6	7	8	9	Average
Λ_*x*_(*G*_1_)	0.2825	0.2825	0.2825	0.2825	0.2825	0.2825	0.2825	0.2825	1.0000	0.3622
${{{{{{{{\mathcal{M}}}}}}}}}_{x}({G}_{1})$	0.2909	0.2867	0.2867	0.2867	0.2867	0.2867	0.2867	0.3074	0.9950	0.3682
Λ_*x*_(*G*_2_)	0.3400	0.3103	0.3103	0.3103	0.3103	0.2321	0.2506	0.5657	0.5657	0.3550
${{{{{{{{\mathcal{M}}}}}}}}}_{x}({G}_{2})$	0.3396	0.3100	0.3100	0.3100	0.3100	0.2379	0.2537	0.5652	0.5914	0.3587

By comparing the single and multilayer node diffusion capacities Λ_*i*_ and ${{{{{{{{\mathcal{M}}}}}}}}}_{i}$, it is possible to know if it is advantageous or not for a node, in terms of diffusion, to be coupled with another network and, by comparing the diffusion capacities Λ(*G*_*L*_) and ${{{{{{{\mathcal{M}}}}}}}}({G}_{L})$, it is possible to know if it is advantageous or not for a layer, in terms of diffusion, to be part of a multilayer system. Node nine in layer *G*_1_ of the structure depicted in Fig. 4A for example, reduces its diffusion-capacity when it is part of the multilayered system.

To explore this relationship between single and multilayer diffusion capacities, we define the relative gain ${{{{{{{\mathcal{G}}}}}}}}$ as the ratio between the multilayer diffusion capacity and the diffusion capacity of the isolated layer. For a node *i* in a layer *G*_*L*_:

7 $${{{{{{{{\mathcal{G}}}}}}}}}_{i}={{{{{{{{\mathcal{M}}}}}}}}}_{i}/{\Lambda }_{i}$$

and

8 $${{{{{{{{\mathcal{G}}}}}}}}}_{L}={{{{{{{\mathcal{M}}}}}}}}({G}_{L})/\Lambda ({G}_{L})$$

In this way, ${{{{{{{{\mathcal{G}}}}}}}}}_{L}$ quantifies the improvement of the DC of layer *L* when it is connected to the other layer in comparison to DC of itself in isolation.

**Fig. 9:**
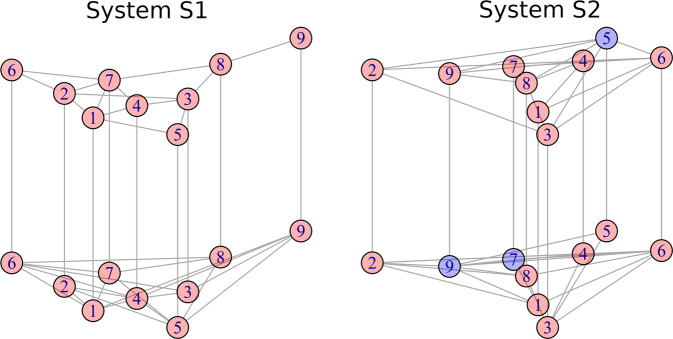
Multiplex network formed by layers *G*_1_ and *G*_2_ connected by weighted links with equal interlayer strength. Nodes in blue/red represent those that decrease/increase their diffusion capacity in the fully coupled multilayer structure, compared to their diffusion capacity in isolation (**A**). Diffusion Capacity Λ of *G*_1_, *G*_2_ and, for different interlayer strengths (logarithmic scale) the multilayer diffusion capacity ${{{{{{{\mathcal{M}}}}}}}}({G}_{1})$ and ${{{{{{{\mathcal{M}}}}}}}}({G}_{2})$ (**B**). Evolution of the Multilayer diffusion Capacity for small interlayer strength values (**C**).

Relative gain is also an important concept for the study of superdiffusion^60,61^, phenomenon in which diffusion processes reach a steady state faster on a multilayer structures than in any of their constitutive layers in isolation. In Fig. 9 are represented two multiplex networks composed by two layers with nine nodes and different connectivity configurations. Systems are built in a way that all red nodes satisfy ${{{{{{{{\mathcal{M}}}}}}}}}_{i} \, > \,{\Lambda }_{i}$ meaning that they are more diffusive in the interconnected system than in the isolated layer, and blue nodes satisfy ${{{{{{{{\mathcal{M}}}}}}}}}_{i} \, < \,{\Lambda }_{i}$ meaning that they are more diffusive in it isolated layer than when it is connected to another structure. To study the emergence of superdiffusion on these structures we use 1000 different random initial conditions and we observe the number of times superdiffusion is developed. Results reveal that superdiffusion is found in 99.8% of cases for *S*1 and in 75.6% of the cases in *S*2, showing the strong influence of the network topology and its dependence on initial conditions. It is interesting to note that, considering the approach based on spectral properties of Laplacian matrices^60^, both structures are considered superdiffusive. This experiment highlight the advantage of the possibility of quantifying DC as it allows the design of structures with specific diffusive requirements and also to investigate the initial conditions in which the structure becomes superdiffusive.

## Supplementary information

Supplementary Information

## Data Availability

The Climate Network Dataset is freely available^42^. The artificial networks data used in the heat model and Kuramoto experiments are provided in the github repository: tischieber/Diffusion-Capacity-of-Single-and-Interconnected-Networks (github.com)^62^.

## Code availability

The source code for computing the Diffusion Capacity and instructions on how to use it are freely available at: tischieber/Diffusion-Capacity-of-Single-and-Interconnected-Networks (github.com)^62^.
