# Supplementary material for: Diffusion capacity of single and interconnected networks
Source: Nat Commun. 2023 Apr 18;14:2217. doi: 10.1038/s41467-023-37323-0 (PMC10113202; doi:10.1038/s41467-023-37323-0)
Supplement: Supplementary file 1 — Supplementary Information [file 41467_2023_37323_MOESM1_ESM.pdf]

SUPPLEMENTARY INFORMATION
Diffusion capacity of single and interconnected networks.

Tiago A. Schieber,[1] Laura C. Carpi,[2,3] Panos M. Pardalos,[4,5]
Cristina Masoller,[6] Albert Díaz-Guilera,[7,8] and Martín G. Ravetti[9,*]

[1] *Departamento de Ciências Administrativas, Universidade Federal de Minas Gerais, Belo Horizonte, MG, Brazil*
[2] *Instituto Nacional de Ciência e Tecnologia, Sistemas Complexos, INCT-SC, CEFET-MG, Belo Horizonte, Brazil*
[3] *Machine Intelligence and Data Science Laboratory (MINDS), Universidade Federal de Minas Gerais, UFMG 31270-000 Belo Horizonte, Brazil*
[4] *Industrial and Systems Engineering, University of Florida, Gainesville, FL, USA*
[5] *Higher School of Economics, Lab LATNA, Nizhny Novgorod, RF*
[6] *Departament de Física, Universitat Politècnica de Catalunya. Rambla St. Nebridi 22, Terrassa 08222, Barcelona, Spain*
[7] *Departament de Física de la Matèria Condensada, Universitat de Barcelona, Barcelona, Spain*
[8] *Universitat de Barcelona Institute of Complex Systems (UBICS), 08028 Barcelona, Spain*
[9] *Departamento de Ciência da Computacão, Universidade Federal de Minas Gerais, Belo Horizonte, MG, Brazil*

(Dated: March 9, 2023)

A. The cumulative Jensen-Shannon divergence (CDD)

Here we consider the Node Distance Distribution in unweighted networks. Thus, for each vertex x, $P_x = (p_1, p_2, \ldots, p_{N-1}, p\infty)$ gives the vector of the density of nodes at a geodesic distance from x. For example, p_1 gives the fraction between the number os nodes at a distance 1 from x and $(N-1)$, being N the number of vertices in the network.

The Jensen-Shannon divergence was successfully used in computing the distance between the intricate patterns of connectivity given by the Node Distance Distributions. However, diffusive systems must carefully consider not only the connectivity patterns but also the appropriate way in which it connects throughout the network.

Consider networks in Figure 1. By comparing the difference in connectivity patterns from node A via the Jensen-Shannon divergence applied to the NDD, we obtain $JS(P_1, P_2) = JS(P_1, P_3) = JS(P_2, P_3)$. This result shows the inefficiency of the divergence in adequately measuring the fundamental difference in connectivity patterns of node A. Note that for a random walker starting from the vertex A, with 1 step it can reach 50% of all possible nodes considering P_1 and 25% for both P_2 and P_3. However, with two steps, both P_1 and P_2 reach 75% of the possible nodes, while the third reaches only 50%. With three steps, a random walker can achieve 100% of the vertices. In this way, it makes sense considering the distance from the first to the second to be smaller than the first to the third.

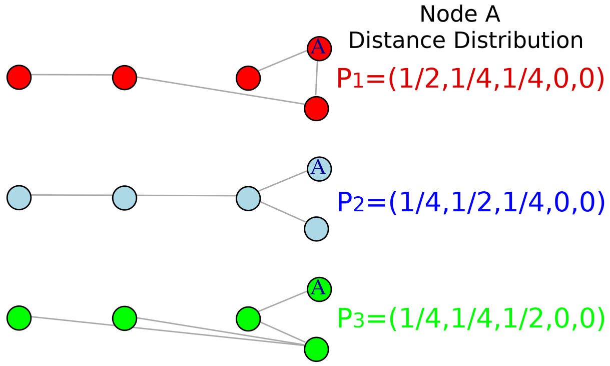

Supplementary Figure 1. Node distance distribution for node A in three different unweighted networks.

To overcome this problem we propose the use of a cumulative JS measure like. Let $P = (p_1, p_2, \ldots, p_N)$ a discrete probability distribution, and let $P^c(x)$ defined as

$$P^c(x) = (\sum_{i=1}^{x} p_i, p_{x+1}, p_{x+2}, \ldots, P_N).$$

We can check that $P^c(1) = P$. For the probability distributions P_1, P_2 and P_3 in Figure 1, for example we have:

$$\begin{array}{ll} P_1^c(2) = (1/2 + 1/4, 1/4, 0, 0) & P_1^c(3) = (1/2 + 1/4 + 1/4, 0, 0) \\ P_2^c(2) = (1/4 + 1/2, 1/4, 0, 0) & P_2^c(3) = (1/4 + 1/2 + 1/4, 0, 0) \\ P_3^c(2) = (1/4 + 1/4, 1/2, 0, 0) & P_1^c(3) = (1/4 + 1/4 + 1/2, 0, 0) \end{array}$$

The interpretation of this cumulative-like distribution is that the first number of the vector $P^c(x)$ represents the fraction of nodes at a distance at most x, the second the fraction of nodes at a distance $x + 1$ and so on.

It is easy to see that

$$JS(P^c(x), Q^c(x)) \leq JS(P^c(y), Q^c(y)) \;\; \forall \;\; x \geq y$$

We define the Cumulative Distance difference between two NDD by the measure:

$$CDD(P, Q) = \sum_x JS(P^c(x), Q^c(x))$$

CDD is a metric and here JS is the square root of the normalized JS divergence.

For the network in Figure 1 we obtain that $CDD(P_1, P_2) < CDD(P_1, P_3)$.

B. The heat model

The heat model consider that each network vertex, i, has an initial chosen temperature, x_i^0. The vertices interact by exchanging heat in order to reach thermal equilibrium with speed proportional to the difference between their temperatures and the weight of the existing links between them. Thus, the higher the weight means the higher rate of heat transfer from one vertex to another. Therefore, a set of N differential equations governing the dynamics of the system are:

$$\frac{dx_i}{dt} = \sum_{j \neq i, j=1}^{N} w_{i,j}(x_j - x_i), \;\; x_i(0) = x^0(i) \;\; \forall \;\; i = 1, \ldots, N$$

which can be rewritten as:

$$\frac{d\mathbf{x}}{dt} = -\mathcal{L} \cdot \mathbf{x}, \qquad \mathbf{x}(0) = \mathbf{x}^0,$$

being $\mathcal{L}$ the graph's Laplacian matrix.

For undirected networks, this system of equations possesses a single solution given by:

$$\mathbf{x}(t) = \mathbf{P} \cdot \mathbf{E}(t) \cdot \mathbf{C},$$

being, $\mathbf{P}$ the matrix whose columns are eigenvectors of $-\mathcal{L}$, $\mathbf{E}(t)$ the diagonal matrix in which the i-th element depends on the i-th eigenvalue of $-\mathcal{L}$, λ_i, given by $e^{\lambda_i t}$ and C a matrix that depends on the initial conditions given by:

$$C = \mathbf{P}^{-1} \cdot \mathbf{x}^0$$

C. Kuramoto oscillators

To understand how diffusion capacity can measure changes in different dynamics associated with complex systems we implemented the Kuramoto model [1], which is characterized by oscillators at each node i, with time-dependent state θ_i, oscillating with an intrinsic natural frequency w_i. If the network consists only of isolated nodes, each vertex will oscillate independently at its natural frequency. However, in a network with adjacency matrix A, the state θ_i of vertex i varies according to the differential equation (1):

$$\dot{\theta}_i = \omega_i + \lambda \sum_j A_{i,j} \sin(\theta_j - \theta_i), \tag{1}$$

where λ is the coupling constant and A is the corresponding adjacency matrix. Now, since a larger absolute value of $\sin(\theta_j - \theta_i)$ implies a larger effect on node i by vertex j through link (j, i), we consider the link weight to be $|\sin(\theta_j - \theta_i)|$.

For this experiment, we use the grid of Figure 1-A, where all oscillators are linked through the same coupling constant ($\lambda = 0.01$). First, we consider the grid with a single oscillator; we choose the same nodes 1 and 2, with a natural frequency $\omega_i = 5$. Figure 2 depicts the results, regardless of the selected node $\Lambda(G)$, tends to increase and return to their initial state, where node 2 that has greater potential Λ_i showing a highest amplitude value.

Figure 2-B shows a different experiment setup. For this case, all nodes are oscillators, and we assign their frequency values with three strategies, namely *Random*, $Max\Lambda_i$, and $Min\Lambda_i$. In *Random*, we attribute random values of natural frequency $\omega_i \sim U(0, 5)$. For $Max\Lambda_i$ and $Min\Lambda_i$, we generate random frequency values using the same distribution and sort them in decreasing order, then sort the nodes using their Λ_i values, decreasing order for $Max\Lambda_i$ and increasing for $Min\Lambda_i$ and assign the ω values to each node list. Figure 2-B depicts results for 100 independent simulations for each case. $Max\Lambda_i$ shows an antipersistent behavior, with a time-series with a Hurst exponent $H = 0.27 \pm 0.01$. On the other hand, $Min\Lambda_i$ show a persistent behavior, $H = 0.84 \pm 0.04$, while a random attribution of frequency seems to generate a random time-series $H = 0.49 \pm 0.06$.

These attribution strategies generate different results. Both $Max\Lambda_i$ and $Min\Lambda_i$ generate systems whose diffusion capacity amplitudes are, on average, more significant than the pure random attribution suggesting that it is possible to intelligently choose, via initial diffusion capacity, elements to increase or decrease the speed with which the system changes. More drastic changes in the system characterize the $Max\Lambda_i$ strategy by either increasing or decreasing the diffusion capacity. It is also interesting to observe each node's role in the dynamics through its local diffusion capacity. In the $Max\Lambda_i$ strategy, node 2 has a diffusion capacity evolution similar to that of the system. At the same time, it does not result in the $Min\Lambda_i$ strategy. Interestingly, this shows that although node 2 has the highest initial diffusion capacity, it cannot describe how the system's dynamics evolve. Information about other vertices is also necessary to understand the system's dynamics. Therefore, the diffusion capacity measure is able to capture the dynamic of the system and it can be use for interventions and further experiments.

D. Diffusion capacity's computational complexity

The diffusion capacity of a single node involves the calculation of its distance to all the others in the network. Although computing shortest paths is a well-known problem, they are often imprac-

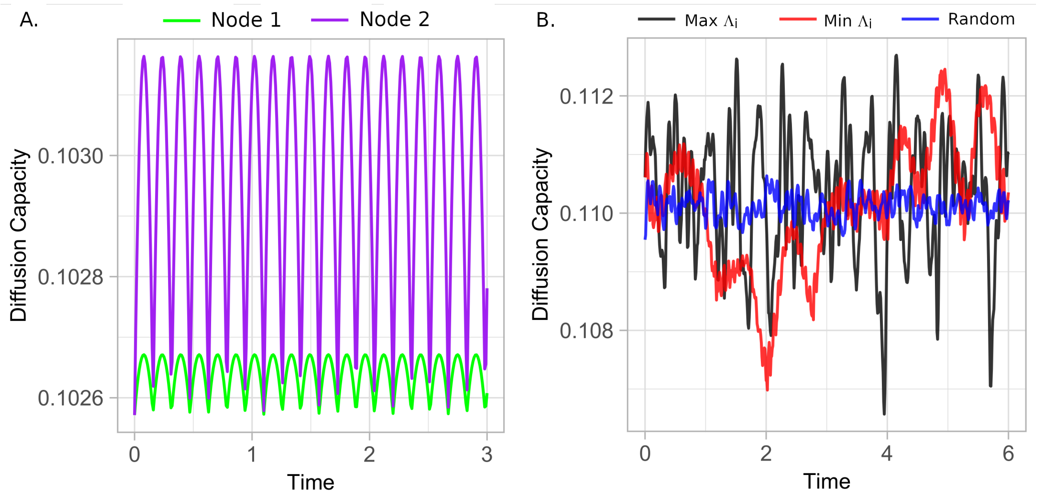

Supplementary Figure 2. Evolution of the Diffusion Capacity in the Kuramoto Model for the grid in Figure 1-A. (A) only one node x (1 or 2) has $w_x = 5$ and all others zero. (B) average values performed on 100 different seeds. Twenty-five natural frequencies were generated uniformly in the interval $(0, 5)$ and distributed among the grid vertices with three different strategies. Randomly (Random), vertices with higher initial diffusion capacity receive higher frequencies (Max Λ_i), and vertices with lower initial diffusion capacity receive higher frequencies (Min Λ_i).

tical for large networks. This section aims to discuss possibilities and the effect that simplifications in computing distances have on diffusion capacity values.

In a network with m links and N vertices, Dijkstra [5] created an algorithm to compute the shortest paths from a single vertex to all the others at the cost of $O(N^2)$ where, for sparse networks, this cost can be reduced to $O(m + NlogN)$. The A^* method [6], on the other hand, is an improvement of Dijkstra's, but in this case, A^* computes the cost of neighboring nodes and then selects the path with the lowest cost to traverse to the destination node. Although A^* enjoys optimality and completeness, the algorithm is not scalable to large networks because the entire network has to be loaded into memory, making the method computationally expensive [7, 8].

Some methods offer heuristics with good approximations. These methods involve intelligently selecting a subset of vertices as reference points and computing the distances from each node to these points of reference. Combining the pre-computed distances makes it possible to estimate the distance between a pair of nodes in a short time. The best-known algorithms involving the combination of Dijkstra, A^* and lower bounds of distances [6, 9–11] have obtained considerable improvements in orders of magnitude and efficiency with a slight loss in accuracy and are therefore candidates for approximations of the diffusion capacity in large networks.

E. Examples of the construction of the weighted node distance distribution of networks in Figure 1 and Figure 3 of the main text.

In layer α, node one posses a direct link to nodes 2 and 3, and two link to node 4, then, the corresponding geodesic distances are $D_g(1, 2) = D_g(1, 3) = 1$, and $D_g(1, 4) = 2$. Now, we compute the minimum weighted distances D_w to each node from node 1. For example, for $D_w(1, 2)$, we have three possibilities, $D_w(1 \to 2) = 1/0.5 = 2$, $D_w(1 \to 3 \to 2) = 1 + 1/2 = 3/2$, and

$D_w(1 \to 5 \to 6 \to 4 \to 3 \to 2) = 1/10 + 1/10 + 1/10 + 1/2 + 1/2 = 13/10$. Then, $D_w(1,2) = 13/10$. In the same way we compute $D_w(1,3) = 8/10$, and $D_w(1,4) = 3/10$, and the corresponding $\Delta_{1,2} = 13/10$, $\Delta_{1,3} = 8/10$, and $\Delta_{1,4} = 3/20$.

$$\mathbf{p}_1^{+}(1) = \frac{1}{3}\max(1-\Delta_{1,2},0) + \frac{1}{3}\max(1-\Delta_{1,3},0) = \frac{1}{3}.\left(0+\frac{2}{10}\right) = \frac{1}{15} = 0.067$$

$$\mathbf{p}_1^{0}(1) = \frac{1}{3}\min\left(\Delta_{1,2},\frac{1}{\Delta_{1,2}}\right) + \frac{1}{3}\min\left(\Delta_{1,3},\frac{1}{\Delta_{1,3}}\right) = \frac{1}{3}.\left(\frac{10}{13}+\frac{8}{10}\right) = 0.523$$

$$\mathbf{p}_1^{-}(1) = \frac{1}{3}\max\left(1-\frac{1}{\Delta_{1,2}},0\right) + \frac{1}{3}\max\left(1-\frac{1}{\Delta_{1,3}},0\right) = 0.077$$

$$\mathbf{p}_1^{+}(2) = \frac{1}{3}\max(1-\Delta_{1,4},0) = \frac{1}{3}0.85 = 0.283$$

$$\mathbf{p}_1^{0}(2) = \frac{1}{3}\min\left(\Delta_{1,4},\frac{1}{\Delta_{1,4}}\right) = \frac{1}{20} = 0.05$$

$$\mathbf{p}_1^{-}(2) = \frac{1}{3}\max\left(1-\frac{1}{\Delta_{1,4}},0\right) = 0$$

$$\mathbf{p}_1^{+}(3) = \mathbf{p}_1^{0}(3) = \mathbf{p}_1^{-}(3) = \mathbf{p}_1(\infty) = 0$$

Then,

$$\mathbb{P}_{1,\mathrm{m}} = [0.067, 0.523, 0.077, 0.284, 0.05, 0, 0, 0, 0, 0] \tag{2}$$

Repeating the process for all nodes and distances, we have the *multilayer distance distributions.*

Now, considering paths that forcibly passes through layer β, $D_w(1,2) = \min\{D_w(1,5) + D_w(5,2), D_w(1,6) + D_w(6,2)\} = 0.8$, $D_w(1,3) = \min\{D_w(1,5) + D_w(5,3), D_w(1,6) + D_w(6,3)\} = 0.3$ and $D_w(1,4) = \min\{D_w(1,5) + D_w(5,4), D_w(1,6) + D_w(6,4)\} = 0.8$. Then, $\Delta_{1,2} = 0.8$, $\Delta_{1,3} = 0.3$, and $\Delta_{1,4} = 0.4$.

$$\mathbf{p}_1^{+}(1) = \frac{1}{3}\max(1-\Delta_{1,2},0) + \frac{1}{3}\max(1-\Delta_{1,3},0) = \frac{1}{3}.\left(\frac{2}{10}+\frac{7}{10}\right) = \frac{3}{10} = 0.3$$

$$\mathbf{p}_1^{0}(1) = \frac{1}{3}\min\left(\Delta_{1,2},\frac{1}{\Delta_{1,2}}\right) + \frac{1}{3}\min\left(\Delta_{1,3},\frac{1}{\Delta_{1,3}}\right) = \frac{1}{3}.\left(\frac{8}{10}+\frac{3}{10}\right) = 0.367$$

$$\mathbf{p}_1^{-}(1) = \frac{1}{3}\max\left(1-\frac{1}{\Delta_{1,2}},0\right) + \frac{1}{3}\max\left(1-\frac{1}{\Delta_{1,3}},0\right) = 0$$

$$\mathbf{p}_1^+(2) = \frac{1}{3}\max\left(1 - \Delta_{1,4}, 0\right) = \frac{1}{3}\frac{6}{10} = 0.2$$

$$\mathbf{p}_1^0(2) = \frac{1}{3}\min\left(\Delta_{1,4}, \frac{1}{\Delta_{1,4}}\right) = \frac{1}{3}\frac{4}{10} = 0.133$$

$$\mathbf{p}_1^-(2) = \frac{1}{3}\max\left(1 - \frac{1}{\Delta_{1,4}}, 0\right) = 0$$

$$\mathbf{p}_1^+(3) = \mathbf{p}_1^0(3) = \mathbf{p}_1^-(3) = \mathbf{p}_1(\infty) = 0$$

$$\mathbb{P}_1^{\beta} = [0.3, 0.367, 0, 0.2, 0.133, 0, 0, 0, 0, 0] \quad (3)$$

Repeating the process for all nodes and distances, we have the distance distribution in which shortest paths are forced to use all layers.

F. Diffusion Capacity and Laplacian Formulation

Laplacian formulation and the diffusion capacity values consider the topology and dynamics, however, the first consists in a global value that represents the diffusion of the entire system, and the second can be computed either associated to a node and also to the system. Diffusion times computed by the system's diffusion capacity are in excellent agreement with those determined by the Laplacian matrix's smallest positive eigenvalue.

It is also essential to consider the behavior of the diffusion capacity in linear or even linearizable dynamical processes. The behavior of these systems is closely related to the second smallest eigenvalue of the Laplacian matrix of the network (λ_2). This eigenvalue, also called algebraic connectivity, has been widely used in the literature to understand, among other things, the asymptotic behavior of the diffusion time in these systems. Importantly, this value is related to the network connectivity and represents global information, losing the local characteristics of the network. For example, $\lambda_2 = 0$ if the network is disconnected, no matters if it has only one disconnected node or if the network has no links.

Figure 3 represents the relationship of diffusion capacity and λ_2 values for 10^5 connected random networks of size 100. Higher values of λ_2 are related to higher values of diffusion capacity and, consequently, lower stability on diffusion times of the dynamical system. There is a strong correlation between λ_2 and the inverse of diffusion capacity -0.87, implying that it is possible to use diffusion capacity to capture the asymptotic behavior of the diffusive process in linear systems. A more interesting result is that the correlation is much stronger when we take less information about the system, such as $d_{thr} = 1$, where the correlation is -0.96 approximately, being an indicator that the diffusion capacity actually can see relationships in the dynamics of the networked system not perceived by the λ_2 alone.

Due to the Abel-Ruffini theorem [2], it is impossible to have a finite-time algorithm that finds all eigenvalues and eigenvectors using only arithmetic and radical operations, and numerical approximations are needed. With an error of the order of 2^{-b}, the time complexity is upper bounded

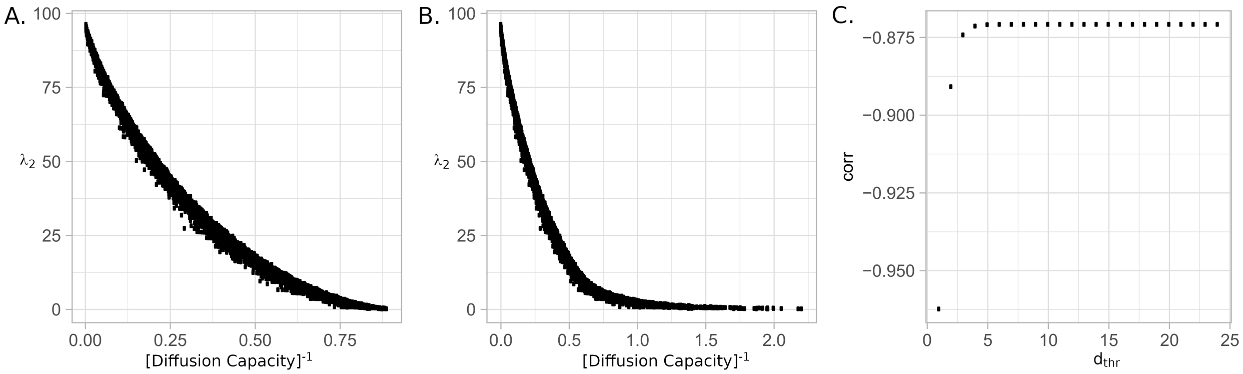

Supplementary Figure 3. Relationship between diffusion capacity and λ_2 values for 10^5 randomly generated connected networks of size 100. (A) shows the relationship for the simplified version of diffusion capacity with $d_{thr} = 1$; (B) the relationship for the full version of diffusion capacity; and (C) the correlation between the inverse of the diffusion capacity and values of λ_2 for different d_{thr}.

by $O(N^3 + (Nlog^2N)log(b))$ [3]. It is also important to note that, when the number of layers is small (less than or equal to five) in a multilayered system, it is possible to derive analytical expressions for eigenvalues of the Laplacian matrix, but for more layers, there are no general algebraic expressions [4].

G. Diffusion capacity and degree correlations between layers.

Let us consider multilayer structures composed by two layers, whose structures are constructed in three different ways. The first one considers that both layers are random, uncorrelated, and undirected graphs, characterized by Poisson distributions with the same mean value. For the second structure, we select replica nodes by degree correlation, that is, nodes with more similar degrees in the different layers, are more likely to be replicas (positively correlated), and for the third structure, replicas nodes possess more dissimilar degrees in the different layers (negatively correlated) [12]. Figure 4 shows the average diffusion capacity values of 100 realizations for the systems above mentioned, for different average degree values. The higher diffusion capacity values correspond to the system constructed by negatively correlated nodes, followed by the structure with the random selection, and finally, the system constructed by the positively selected nodes. As shown in Figure 4-A, this difference is higher for low average degrees, becoming more similar as the average degrees increase. The same behavior is valid for the average diffusion capacity values for isolated layers; however, the difference is more accentuated. Figure 4-B shows the relative gain $\mathcal{G}$. In the first case, we observe that, as the average degree of the layers increases, $\mathcal{G}$ increases due to the lack of mutually connected components, as the shortest paths correspond, in the majority, to interlayer links. This behavior remains until mutually connected components emerge, revealing a phase transition, and determining the value of the average degree c from which $\mathcal{G}$ stars to decrease. This result is consistent with findings in [13], where a hybrid phase transition with a discontinuity in the number of driver nodes is observed for the same c value. The relative gain $\mathcal{G}$ of the negatively correlated system possess the highest value, followed by uncorrelated system and then by the positively correlated one that shows two local maxima values, one at a small average degree value c and a more pronounced one at an intermediate c value. All systems possess similar

$\mathcal{G}$ values for intermediate to high c values. Additional figures of this experiment can be found in section E of the SI. An interesting example regarding diffusion capacity and node heterogeneity in multilayer networks is presented in section F of the SI.

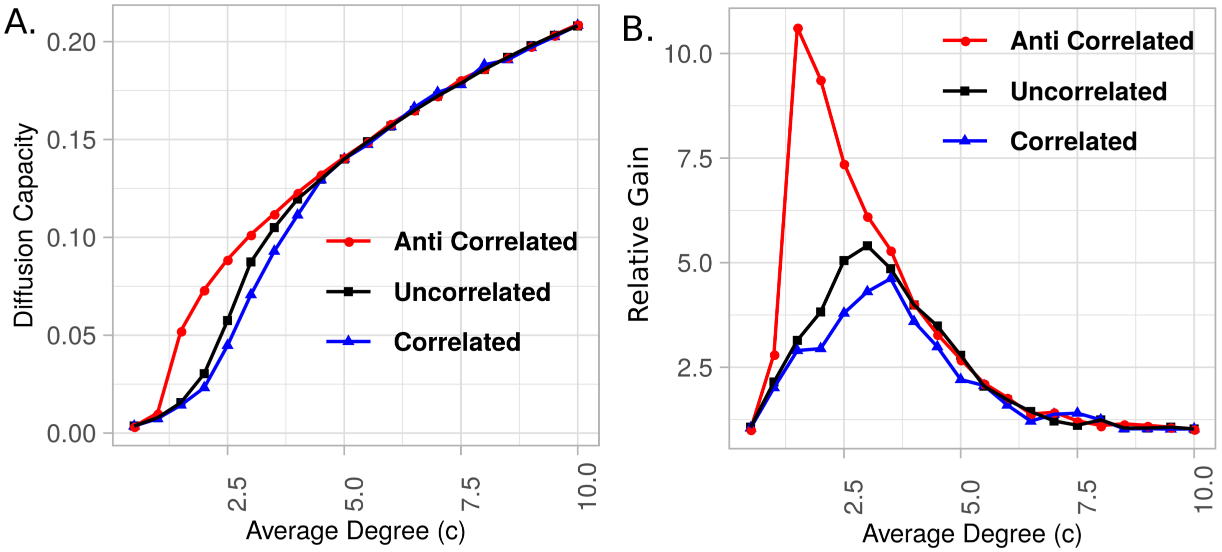

Supplementary Figure 4. Average diffusion capacity values (A) and relative gain $\mathcal{G}$ values (B) of 100 realization of duplex networks considering three different inter-layers connectivity strategies (see section F of the SI for the corresponding heat maps of negatively correlated, uncorrelated and positively correlated layers).

H. Diffusion Capacity in a multilayer brain network

In this section we study the involvement of the central nervous system via electroencephalography (EEG) of the 9-layer network constructed by Huang et al. [14], considering the three types of patients: complete deafness (D), tinnitus (T), and normal controls (N). Tinnitus arises as an uncomfortable sound inside the ear that occurs when it seems to identify sound impulses without them being sent externally. In some cases, it causes discomfort and can affect sleep, concentration, and emotional balance, resulting in a significant impact on people's daily activities [15, 16].

We construct a structure of 9 layers corresponding to the frequencies Delta (1-4Hz), Theta (4-8Hz), Alpha1 (8-10Hz), Alpha2 (10-12Hz), Beta1 (13-18Hz), Beta2 (18-21Hz), Beta3 (21-30Hz), Gamma1 (30.5-45Hz) and Gamma2 (55-70Hz).

Figure 5 shows average values of diffusion capacity and relative gain for different phase constants α considering the averages of 1000 random considering the FitzHugh-Nagumo model in a network with two layers of coupled neurons, as in [17]. Interesting to note that the Tinnitus system has a higher diffusion capacity than the other ones but, on the other hand, a lower relative gain in the multilayered system. The opposite is true for patients with complete deafness. By consider brain regions sets of vertices, it is interesting to observe that the Central Frontal (CF), Left Posterior Medial (LPM), and Right Posterior Medial (RPM) regions have a higher diffusion capacity in Tinnitus patients than when compared to the others. These findings corroborate recent results that the ventromedial prefrontal cortex is considered a critical region involved in tinnitus, and the

precuneus is a possible invariant marker of long-term tinnitus [16, 18]. Regarding the increased relative gain in deaf patients, the Central Frontal (CF) and the Right Anterior Lateral (RAL) showed the highest relative gains in the system. In contrast, Left Anterior Lateral (LAL) showed the lowest being an intriguing result since a crucial region for speech processing is more significant in the left hemisphere than in the right, giving the left hemispheric an advantage in speech perception reflected by the left faster neurophysiological processing [19]. One possible hypothesis is that this region in the deaf patients does not need to have an increased relative gain when compared to the others because it may not receive stimulus in this patients.

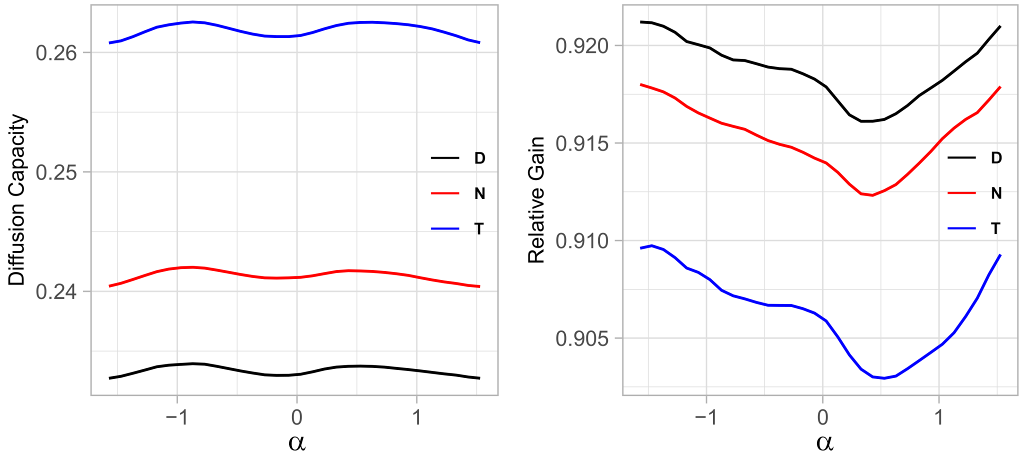

Supplementary Figure 5. Average Diffusion Capacity values (A) and Relative Gain for different phase constants α for Tinnitus (T), complete deafness (D) and normal (N) networks.

SUPPLEMENTARY REFERENCES

* martin@dcc.ufmg.br

[1] Kuramoto, Y. Self-entrainment of a population of coupled nonlinear oscillators. In Araki, H. (ed.) *International Symposium on Mathematical Problems in Theoretical Physics, Lecture Notes in Physics, Vol. 39,*, 420–422 (Springer, New York, NY, USA, 1975).

[2] Abel, N. H. *Mémoire sur les équations algébriques, où l'on démontre l'impossibilité de la résolution de l'équation générale du cinquième degré* (Christiania, 1824). Repr. in *Œuvres complètes*, éd. L. Sylow, S. Lie, t. 1, Christiania : Grøndahl, p.28-33.

[3] Pan, V. Y. & Chen, Z. Q. The complexity of the matrix eigenproblem. In *Proceedings of the Thirty-First Annual ACM Symposium on Theory of Computing*, STOC '99, 507–516 (Association for Computing Machinery, New York, NY, USA, 1999). URL `https://doi.org/10.1145/301250.301389`.

[4] Solé-Ribalta, A. *et al.* Spectral properties of the laplacian of multiplex networks. *Physical review. E, Statistical, nonlinear, and soft matter physics* **88 3**, 032807 (2013).

[5] Dijkstra, E. W. A note on two problems in connexion with graphs. *Numerische Mathematik* **1**, 269–271 (1959).

[6] Goldberg, A., Kaplan, H. & Werneck, R. Reach for a^*: Efficient point-to-point shortest path algorithms. Tech. Rep. MSR-TR-2005-132 (2006). URL `https://www.microsoft.com/en-us/research/publication/reach-for-a-efficient-point-to-point-shortest-path-algorithms/`. Technical Report for CLASSiC FP7 European project.

[7] Potamias, M., Bonchi, F., Castillo, C. & Gionis, A. Fast shortest path distance estimation in large networks. In *Proceedings of the 18th ACM Conference on Information and Knowledge Management*, CIKM '09, 867–876 (Association for Computing Machinery, New York, NY, USA, 2009). URL `https://doi.org/10.1145/1645953.1646063`.

[8] Mensah, D. N. A., Gao, H. & Yang, L. W. Approximation algorithm for shortest path in large social networks. *Algorithms* **13** (2020). URL `https://www.mdpi.com/1999-4893/13/2/36`.

[9] Ikeda, T. *et al.* A fast algorithm for finding better routes by ai search techniques. *Proceedings of VNIS'94 - 1994 Vehicle Navigation and Information Systems Conference* 291–296 (1994).

[10] Goldberg, A. V. & Harrelson, C. Computing the shortest path: A search meets graph theory. In *Proceedings of the Sixteenth Annual ACM-SIAM Symposium on Discrete Algorithms*, SODA '05, 156–165 (Society for Industrial and Applied Mathematics, USA, 2005).

[11] Xiao, Y., Wu, W., Pei, J., Wang, W. & He, Z. Efficiently indexing shortest paths by exploiting symmetry in graphs. In *Proceedings of the 12th International Conference on Extending Database Technology: Advances in Database Technology*, EDBT '09, 493–504 (Association for Computing Machinery, New York, NY, USA, 2009). URL `https://doi.org/10.1145/1516360.1516418`.

[12] Nicosia, V. & Latora, V. Measuring and modeling correlations in multiplex networks. *Physical Review E* **92**, 032805– (2015). URL `https://link.aps.org/doi/10.1103/PhysRevE.92.032805`.

[13] Menichetti, G., Dall'Asta, L. & Bianconi, G. Control of multilayer networks. *Scientific Reports* **6**, 20706 (2016).

[14] Huang, L., Wang, C.-D. & Chao, H.-Y. Hm-modularity: A harmonic motif modularity approach for multi-layer network community detection. *IEEE Transactions on Knowledge and Data Engineering* **33**, 2520–2533 (2021).

[15] Chan, Y. Tinnitus: etiology, classification, characteristics, and treatment. *Discov Med.* **8** (2009).

[16] Ueyama, T. *et al.* Brain regions responsible for tinnitus distress and loudness: A resting-state fmri study. *PLOS ONE* **8**, 1–12 (2013). URL `https://doi.org/10.1371/journal.pone.0067778`.

[17] Kang, L., Tian, C., Huo, S. & Liu, Z. A two-layered brain network model and its chimera state. *Scientific Reports* **9**, 2045–2322 (2019).

[18] Schmidt, S. A., Carpenter-Thompson, J. & Husain, F. T. Connectivity of precuneus to the default mode and dorsal attention networks: A possible invariant marker of long-term tinnitus. *Neuroimage Clin.* **22**, 196–204 (2017).

[19] Sebastian, O. *et al.* Neurite architecture of the planum temporale predicts neurophysiological processing of auditory speech. *Science Advances* **4** (2021).
